# Neuroprotective Herbs Associated with Parkinson’s and Alzheimer’s Disease

**DOI:** 10.3390/nu18030439

**Published:** 2026-01-29

**Authors:** Georgiana Smaranda Marțiș, Rodica Ana Ungur, Anamaria Pop, Evelina Maria Bordean, Claudia Pașca, Ileana Monica Borda

**Affiliations:** 1 Faculty of Food Science and Technology, University of Agricultural Sciences and Veterinary Medicine Cluj-Napoca, 64 Calea Floresti, 400509 Cluj-Napoca, Romania; georgiana.martis@usamvcluj.ro (G.S.M.); anamaria.pop@usamvcluj.ro (A.P.); 2 Department of Medical Specialties, Faculty of Medicine, “Iuliu-Hațieganu” University of Medicine and Pharmacy, 8 Victor Babeș Street, 400012 Cluj-Napoca, Romania; monica.borda@umfcluj.ro; 3 Raluca Ripan Technical College, 21th Bistritei, 400430 Cluj-Napoca, Romania; bordeanmariaevelina@gmail.com; 4 Department of Apiculture and Sericulture, Faculty of Animal Science and Biotechnologies, University of Agricultural Sciences and Veterinary Medicine Cluj-Napoca, Calea Mănăştur 3–5, 400372 Cluj-Napoca, Romania; claudia.pasca@usamvcluj.ro

**Keywords:** Parkinson’s disease, Alzheimer’s disease, plant bioactive, oxidative stress, synaptic protection, natural compounds

## Abstract

There is currently no treatment for Parkinson’s (PD) and Alzheimer’s (AD) diseases, and medications that target the blockage of amyloid plaque cascades appear to be the most promising for preventing these diseases. However, it is believed that consuming natural antioxidants, particularly phytochemicals such as phenolic compounds, may help the treatment process for neurodegenerative illnesses. Phenolic substances such as phenolic acids, polyphenols, and flavonoids have been shown to have antioxidant properties in plants and are thought to have a similar impact in humans. This review provides an analysis of the current landscape of PD and AD pathophysiology, paying particular attention to phytochemical-based therapeutic, preventive, and management strategies using disclosed herb candidates in in vivo/vitro studies. We also highlight the herb-derived components that have recently been identified for their effects in the treatment of PD/AD to provide a review and perspectives for the development of the next generation of drugs and preparations for the treatment of PD/AD.

## 1. Introduction

Globally, the prevalence of Alzheimer’s disease (AD) and Parkinson’s disease (PD) is increasing, and there is an urgent need for potent pharmaceutical treatments. There is a significant amount of proof that oxidative stress has a role in the onset and course of these two neurodegenerative diseases (NDDs). Overproduction of reactive oxygen species (ROS) and reactive nitrogen species (RNS), mitochondrial dysfunction, neuroinflammation, and neurodegeneration are all caused by oxidative stress, which is also a key factor in the pathogenic processes of these two diseases. Antioxidants are thus interesting treatment possibilities for AD and PD [1].

Phytochemicals may be linked to traditional dementia treatments, serve as precursors for synthetic analogs, or be crucial in the development of new medications. The neuroprotective properties of substances like terpenoids and flavonoids have been reviewed, indicating their possible applicability to conventional dementia treatments [2]. However, extensive clinical evidence is lacking or contentious, and further clinical trials are needed to better evaluate their therapeutic or preventative potential in dementia. Oxidative stress causes abnormal proteins (such A and a-synuclein), neuroinflammation, mitochondrial malfunction, and neuronal death (particularly cholinergic and dopaminergic neurons). Furthermore, some flavonoid metabolites (particularly specific products of bacterial transformation) have piqued the interest of academics and pharmacists due to their potent bioactivities [3]. Anthocyanins and phenolic acid metabolites (including 4-Hydroybenzoic acid, protocatechuic acid, gallic acid, 3-O-methygallic acid, vanillic acid, and syringic acid) have been shown to be antioxidants, exhibit anti-neuroinflammation, interfere with protein aggregation, and have good neuroprotective properties [4].

The objective of this study is to summarize the existing evidence on the therapeutic potential of herbs’ phytochemicals in preventing and alleviating symptoms of Alzheimer and Parkinson. The physiological part of herbs that is used, natural component, method and concentration of the extract, type of study (in vivo/in vitro), and mechanism of action assessments were also evaluated to achieve an overall medical preventive and management strategies for Alzheimer’s and Parkinson’s diseases.

## 2. Parkinson’s Disease

### 2.1. Oxidative Stress and Neuronal Damage in Parkinson Treatment

Parkinson’s disease manifests as both motor and non-motor symptoms due to the progressive degeneration of dopaminergic neurons in the substantia nigra, which is linked to α-synuclein aggregation, oxidative stress, mitochondrial dysfunction, neuroinflammation, and impaired protein degradation [5,6].

Among the multiple pathogenic mechanisms implicated in PD, oxidative stress and the resulting neuronal damage occupy a central position. Mitochondrial dysfunction, excessive production of reactive oxygen species (ROS), impaired antioxidant defenses, lipid peroxidation, and activation of apoptotic signaling cascades collectively contribute to dopaminergic neurodegeneration. Consequently, therapeutic strategies that target oxidative stress and its downstream effects have gained considerable attention, particularly through the investigation of plant-derived compounds and extracts with antioxidant and neuroprotective properties.

A substantial body of experimental evidence demonstrates that oxidative stress is a key mediator of dopaminergic cell death in PD models. Neurotoxins such as 6-hydroxydopamine (6-OHDA), rotenone, MPTP, and MPP^+^ are widely used to reproduce PD-like pathology, precisely because they induce oxidative damage, mitochondrial impairment, and apoptosis. Within this context, *Artemisia absinthium* has emerged as a promising candidate. Rashidi et al. [7] demonstrated that *A. absinthium* extract significantly attenuated 6-OHDA-induced ROS production and apoptosis in SH-SY5Y cells. The extract reduced malondialdehyde (MDA), a marker of lipid peroxidation, while restoring glutathione (GSH) levels and superoxide dismutase (SOD) activity. These findings underscore the ability of *A. absinthium* to reinforce endogenous antioxidant defenses and limit oxidative neuronal injury, suggesting its potential as an adjunct to conventional PD therapies.

*Ginkgo biloba* is one of the most extensively studied botanical agents in relation to neurodegeneration. Although early clinical observations were limited to case reports, such as the report by Conrad [8], which described marked symptomatic improvement in a PD patient receiving *Ginkgo biloba* supplementation, subsequent experimental studies provided mechanistic insights. Zhang et al. [9] showed that *Ginkgo biloba* extract (EGb) mitigated oxidative damage in a sodium fluoride-exposed rat model, enhancing antioxidant enzyme activities (SOD and GSH-Px), reducing MDA levels, and modulating apoptotic regulators by increasing the Bcl-2/Bax ratio and decreasing caspase-3 activation. In PD-relevant models, Rojas et al. [10] demonstrated that EGb761 prevented MPP^+^-induced dysregulation of copper homeostasis in key brain regions, including the striatum, midbrain, and hippocampus. Given the role of metal-catalyzed oxidative reactions in PD pathogenesis, this ability to stabilize the copper distribution further supports the antioxidant and neuroprotective profile of *Ginkgo biloba*.

*Curcuma longa* and its principal bioactive compound, curcumin, represent another extensively investigated intervention targeting oxidative stress-mediated neuronal damage. In vitro studies using SH-SY5Y cells exposed to salsolinol revealed that *C. longa* extract reduced mitochondrial ROS production, suppressed apoptotic signaling, and downregulated p53, Bax, and caspase-3 expression [11]. Complementary in vivo studies reinforced these findings. Cui et al. [12] demonstrated that curcumin alleviated motor deficits and preserved tyrosine hydroxylase (TH) expression in rotenone-treated rats, concomitant with increased GSH levels and reduced ROS and MDA content. Importantly, this neuroprotection was mechanistically linked to activation of the Akt/Nrf2 signaling pathway, a master regulator of cellular antioxidant responses.

Saffron (*Crocus sativus* L.) and its bioactive constituent crocin have also demonstrated robust antioxidant-mediated neuroprotection. In a *Drosophila* model of Parkinsonism, Rao et al. [13] showed that saffron methanolic extract and crocin reduced oxidative stress markers, restored GSH and thiol levels, improved mitochondrial enzyme activities, and normalized dopamine levels. These antioxidant effects translated into improved locomotor function and increased lifespan. In mammalian models, saffron preconditioning significantly protected dopaminergic neurons against MPTP-induced toxicity, preserving TH-positive cells in the substantia nigra and retina [14]. Transcriptomic analyses further revealed that saffron modulated multiple neuroprotective pathways, including those related to redox regulation and apoptosis [15]. These findings suggest that saffron exerts both acute antioxidant effects and longer-term genomic adaptations that enhance neuronal resilience to oxidative stress.

*Carthamus tinctorius* (safflower), particularly in the form of standardized safflower flavonoid extract (SAFE), has been shown to attenuate oxidative stress and neuronal damage in PD models. Ren et al. [16] reported that SAFE improved behavioral performance in 6-OHDA-lesioned rats while suppressing α-synuclein overexpression and reactive astrogliosis. Ablat et al. [17] further demonstrated that SAFE restored dopaminergic markers, including TH and dopamine transporter expression, and normalized dopamine levels. The modulation of extracellular space diffusion parameters observed via MRI suggests that antioxidant protection may extend to preserving neuronal and glial integrity.

Additional botanical agents further reinforce the centrality of oxidative stress modulation in neuroprotection. Carnosic acid from *Rosmarinus officinalis* reduced lipid peroxidation, restored GSH levels, inhibited stress-activated kinases (JNK and p38), and prevented apoptosis in 6-OHDA models [18]. Isoliquiritigenin from *Glycyrrhiza uralensis* attenuated ROS and nitric oxide production while stabilizing mitochondrial membrane potential via PI3K/Akt signaling [19]. *Nigella sativa* and its bioactive compound thymoquinone consistently reduced lipid peroxidation, restored antioxidant enzyme activity, protected nigral neurons, and improved motor outcomes in several PD models [20,21]. Similarly, combined extracts of *Zingiber officinale* demonstrated reductions in oxidative damage and monoamine oxidase-B activity, linking antioxidant action with improved dopaminergic function [22].

### 2.2. Phytochemical-Based Therapeutic Strategy for Parkinson Treatment

Major plants studied for Parkinson’s disease are summarized in Table 1. It summarizes fourteen plants that focus on the physiological part used, type of extract/dose, type of study, and positive mechanism of action on Parkinson’s disease.

One of the most extensively studied phytochemicals in PD research is curcumin, derived from *Curcuma longa*. Complementary findings in 6-OHDA-induced Parkinsonian rats showed that curcumin preserved dopaminergic markers, normalized antioxidant enzymes, and improved behavioral outcomes [23]. These results suggest that curcumin functions as a disease-modifying agent rather than a simple antioxidant scavenger.

Similarly, *Ginkgo biloba* extracts (EGb) have demonstrated neuroprotective effects across several experimental paradigms. Moreover, EGb761 prevented neurotoxin-induced dysregulation of copper homeostasis in PD models, addressing metal-mediated oxidative stress—a recognized contributor to dopaminergic neurodegeneration [10].

Saffron (*Crocus sativus* L.) represents another phytochemical intervention with strong experimental support. Both saffron extracts and crocin significantly protected dopaminergic neurons in *Drosophila* and mammalian PD models by reducing oxidative stress, restoring mitochondrial function, and preserving dopamine levels [14]. Transcriptomic analyses further revealed that saffron modulates multiple neuroprotective pathways, including those related to apoptosis, inflammation, and redox balance [15]. These pleiotropic effects underscore saffron’s potential as a supplementary therapeutic agent in PD.

Flavonoid-rich preparations such as standardized safflower flavonoid extract (SAFE) from *Carthamus tinctorius* also exhibit therapeutic promise. SAFE improved motor behavior, restored dopaminergic neurotransmission, suppressed α-synuclein overexpression, and attenuated reactive astrogliosis in toxin-induced PD models [16,17]. Notably, neuroimaging-based assessments indicated that SAFE partially normalized extracellular space diffusion parameters, suggesting preservation of neuronal and glial architectures.

Adjunctive phytochemical strategies are particularly relevant in addressing complications of long-term L-DOPA therapy. Extracts from *Gynostemma pentaphyllum* significantly attenuated L-DOPA-induced dyskinesia in 6-OHDA-lesioned rats by downregulating ΔFosB expression and ERK1/2 phosphorylation, without diminishing the therapeutic efficacy of L-DOPA itself [24]. In parallel, *Mucuna pruriens*, a natural source of L-DOPA enriched with antioxidant compounds, demonstrated favorable clinical outcomes, including prolonged “on” time and reduced adverse effects, with no reported dyskinesia [25].

Regulation of neuroprotective proteins represents an additional therapeutic axis. Cinnamon (*Cinnamomum verum*) and its metabolite sodium benzoate upregulated DJ-1 and Parkin—essential proteins for mitochondrial quality control—while suppressing inflammatory nitric oxide signaling in both cellular and animal PD models [26,27]. Likewise, *Nigella sativa* and its active compound thymoquinone improved motor rigidity, reduced lipid peroxidation, and preserved dopaminergic neurons in multiple PD models [20,21].

**Table 1:** Positive pharmacological activity of bioactive plant compounds on Parkinson’s disease.

Herb	Physiological Part Used	Major Component	Country	Type of Extract/Dose	Method of Obtainment	Type of Study	Mechanism of Action	References
Black cumin (*Nigella sativa* L.)	Seeds	Thymoquinone	India	Standardized ethanolic extract; doses of 200 and 400 mg/kg body weight for 21 days; administered orally, once daily.	Extract was procured from Amsar Private Limited, 47, Laxmibai Nagar, Fort, Industrial estate, Indore 452006, Madhya Pradesh, India	In vivo	*Nigella sativa* significantly reduces cataleptic scores, showing its anti-cataleptic activity.	[20]
		Antioxidants (not specified)	Iran	Hydroalcoholic extract; doses of 50, 100, and 200 mg/kg were administered orally to male mice for 12 days, once daily.	Black seeds were cleaned, dried, mechanically powdered, extracted with 70% ethanol, and dried with a rotary evaporator to render the extract alcohol-free and kept in refrigerator at 4 °C until used.	In vivo	Black seed can improve muscle rigidity very well compared to levodopa.	[28]
		Thymoquinone	Iran	Standardized solid extract thymoquinone; doses of 5 and/or 10 mg/kg three times at an interval of 24 h. Administered intraperitoneally, once daily.	Sigma Chemical, St. Louis, MO, USA; purity > 97%	In vivo	Thymoquinone at a dose of 10 mg/kg significantly decreases apomorphine-induced rotations, attenuates loss of SNC neurons, and lowers midbrain level of malondialdehyde in 6-OHDA-lesioned rats.	[21]
Cinnamon (*Cinnamonum verum*)	Bown bark of cinnamon tree	Sodium benzoate	Chicago	Standardized extract 500 μM of sodium benzoate; orally, once daily.	-	In vivo	Sodium benzoate increases the level of DJ-1 (neuroprotective protein) by modulating the mevalonate pathway and that p21ras, but not p21rac, is involved in the upregulation of DJ-1.	[26]
	Brown bark of cinnamon tree/Cinnamon powder	Cinnamaldehyde and sodium benzoate	California, UAS	Cinnamon extract; 100 μL cinnamon–MC mixture; orally, once daily (7 days).	Ground cinnamon was solubilized in 0.5% methylcellulose	In vivo	Dopaminergic neuronal protection, normalized striatal neurotransmitters, and improved motor functions by cinnamon in MPTP-intoxicated mice.	[27]
Ginger (*Zingiber officinale* L.) and Purple nut sedge (*Cyperus rotundus* L.)	Rhizome	Quercetin	Thailand	Ethanolic extract; 100, 200, and 300 mg·kg^−1^ BW for 14 days after oxidopamine injection; orally, once daily.	The plant materials were prepared as 95% ethanolic extract. A ratio of ethanolic extract of aerial part of *C. rotundus* and rhizome of *Z. officinale* which provided the highest potential to enhance memory (1:5) was prepared. The percentage yields of the *C. rotundus* and *Z. officinale* extracts were 7.41% and 10.48%, respectively.	In vivo	Increased spatial memory but decreased neurodegeneration, malondialdehyde level, and acetylcholinesterase activity in hippocampus. CP1 is the potential functional food against Parkinson’s disease.	[22]
Ginkgo (*Ginkgo biloba* L.)	Leaves	Terpene trilactones and flavonoid glycosides	USA	Standardized extract; 40 mg three times a day, over a 6-week period; orally, once daily.	24% ginkgoflavonglycosides, 6% terpene lactones, and 2% bilobalide	In vivo	Falling episodes dramatically decrease, but tremors improved by 80% to 90%.	[8]
		Flavonoids and terpenoids	Mexico	Standardized extract—EGb761/10 mg/kg daily for 17 days.	EGb 761 [Ginkgo biloba extract EGb 761, Rökan, Tanakan, Tebonin]	In vivo	The protective effect of EGb761 against MPP neurotoxicity may be due in part to the regulation of copper homeostasis in the brain.	[10]
Ginseng (*Panax ginseng*)	Root	Ginsenosides	Chine	Aqueous extract; 0.013 g/kg body weight per day, 0.026 g/kg body weight per day, and 0.039 g/kg body/28 days.	Ginseng was extracted with neutral aqueous solution (pH 7.0 ± 0.1; ratio of ginseng powder to solvent 1:10) for three times for 12 h at 4 °C. Using centrifugal supernatant fluid filtration, we obtained the samples after vacuum freeze drying.	In vivo (animal modal)	Reduced loss of dopaminergic neurons, improved motor coordination, reduced neuroinflammation	[29]
Jiaogulan (*Gynostemma pentaphyllum*)	Leaves	Gypenosides	China	Standardized gypenosides (purity > 99%); Gypenosides: 25 and 50 mg/kg). Ethanol extract: 50 mg/kg, orally, once daily for 22 days.	Gypenosides were purchased from Ankang Dongke Maidisen Nature Pharmaceutical Co. (Xi’an, China) Ethanol extract: The air-dried leaves of Jiaogulan (1 kg) were extracted with ethanol (80%, *v*/*v*), and the ethanol extracts were evaporated to dryness under reduced pressure and temperature (97.2 g, yield, 9.7%, *w*/*w*).	In vivo	Gypenosides and ethanol extract of Jiaogulan show anxiolytic effects on affective disorders in 1-methyl-4-phenyl-1,2,3,6-tetrahydropyridine-lesioned mouse model of Parkinson’s disease.	[24]
Licorice (*Glycyrrhiza uralensis*)	Roots	Isoliquiritigenin	California, USA	Aqueous extract/1; 0.5 µM; added directly to cell culture medium; single pretreatment, applied 1 h before oxidopamine exposure.	A total of 100 g of licorice root powder was decocted for 1 h with 1 L of reverse-osmotic water 3 times. The aqueous extract of licorice was filtered and concentrated with a vacuum rotary evaporator and then lyophilized. The lyophilized extract was dissolved in water a concentration of 200 mg/mL, applied to Sephadex LH-20, and then eluted by gradient ethanol concentrations (0%, 5%, 20%, 40%, 60%, 80%, and 100%).	In vitro	Extract mediated neuroprotection in dopaminergic neurons and was involved in attenuation of oxidative stress and mitochondria-related apoptotic cell death, and in modulation of brain-derived neurotrophic factor levels and the PI3K-Akt/PKB signaling pathways.	[19]
Rosemary (*Rosmarinus officinalis* L.)	Leaves	Carnosic acid	California, USA	Standardized extract was obtained from A. G. Scientific, Inc. (San Diego, CA, USA); dose of 20 mg/kg body weight; orally, once daily, pretreated for 3 weeks before the oxidopamine lesion was induced.	Carnosic acid was dissolved in 0.5% sodium carboxy methyl cellulose.	In vivo/in vitro	Carnosic acid reduced the apomorphine-caused rotation in 6-OHDA-stimulated rats. Significant protection against lipid peroxidation and glutathione peroxidase reduction was observed in the 6-OHDA rats pretreated with carnosic acid.	[18]
Safflower (*Carthamus tinctorius* L.)	Flower petals	Standardized flavonoid extract of safflower/Kaempferol 3-*O*-rutinoside (K3R) and anhydrosafflor yellow (AYB)	China	Ethanolic extract/35 mg/kg/day, and 70 mg/kg/day; orally, once daily. In vitro marker compounds were added to cell culture medium of Pc12 cells/50, 100, and 200 µM used for pretreatment.	The dried flower petals were twice extracted with 50% EtOH at a ratio of 1:8. The collective extract was concentrated in a rotary evaporator and chromatographed on an AB-8 macropore resin column using gradient elution, with 15%, 30%, 50%, 70%, and 95% EtOH. Extract from safflower was isolated and purified with macropore resin, and the quality standard was preliminarily established.	In vitro/in vivo	Safflower extract may partially restore dopaminergic neuron numbers. Safflower treatment could partially inhibit the changes in diffusion in the extracellular space, which might provide some information about neuronal loss and astrocyte activation.	[16]
	Herbs	kaempferol 3-*O*-rutinoside and anhydrosafflor yellow B	China	Ethanolic extract standardized safflower flavonoid extract; 35 or 70 mg/kg/day; orally by gavage, once daily for 24 days.	A total of 8kg of safflower was soaked in 50% ethanol (8 L/kg) for 2–3 h, followed by heat extraction under reflux twice, with 1 h for each cycle. The extracts were combined, concentrated in vacuum, and diluted with de-ionized water to produce a thin extract at a concentration of 1 g/mL. The macroporous resin separation method was used to elute the extract with 10%, 30%, 50%, and 95% ethanol.	In vivo	In this study, kaempferol 3-O-rutinoside and anhydrosafflor yellow B comprised the quality standard of the safflower extract and demonstrated the neuroprotective properties of flavonoids.	[17]
Saffron (*Crocus sativus* L.)	Stigmats	carotenoids, monoterpene aldehydes, monoterpenoids, isophorones, and flavonoids	India	Methanolic extract; 0.05% and 0.1%; administered via enriched diet medium; frequency: continuously provided in the diet; 7 days.	Saffron was finely cut, dried, and macerated with methanol (1:50 *w*/*v*) in dark under continuous stirring for 72 h. The extract was centrifuged and filtered through 0.2 mm filter and evaporated to dryness using a rotary evaporator. The dry residue was stored at 20 °C until use. The final yield was 25%.	In vitro	Due to its antioxidant action, saffron may be exploited as a supplementary therapeutic agent in PD and other oxidative stress-mediated neurodegenerative conditions.	[13]
		crocetin	Australia	Aqueous extract/50 mg/kg; orally, daily, 7 consecutive days.	Specifically, saffron stigmata were weighed and ground with a mortar and pestle and diluted to 0.01% (*w*/*v*) in standard rodent drinking water; the aqueous extract was renewed every 2–3 days.	In vivo	The apparent biphasic nature of the dose–response relationship between saffron and measures of neuroprotection, together with the stress-inducible nature of many of the upregulated genes and pathways, lend credence to the idea that saffron, like various other phytochemicals, is a hormetic stimulus, with functions beyond its strong antioxidant capacity.	[15]
		Crocin, safranal	Australia	Aqueous extract/2 mL/day/oral/5 consecutive days.	A total of 10 mg of saffron was put into 100 mL water overnight to allow the water-soluble components (including crocin and safranal) to dissolve.	In vivo	Saffron pre-conditioning mitigated the reduction, with pre-conditioned 1-methyl-4-phenyl-1,2,3,6-tetrahydropyridine-injected mice having SNc and retinal TH+ cell numbers close to control levels, significantly (25–35%) higher than in non-conditioned MPTP-injected mice.	[14]
Turmeric (*Curcuma longa*)	Dried roots of the rhizome	Curcumin	India	Curcumin; 80 mg/kg body; oral gavage, once daily; 21 consecutive days.	Standardized extract.	In vivo	Curcumin significantly decreased the apomorphine-induced circling in rats and attenuated damage of substantia nigra dopaminergic neurons induced by 6-OHDA in rats.	[23]
	Rhizome	Curcuminoid	China	Aqueous extract; 0.001, 0.01, 0.05, 0.1, 0.,2 and 0.4 mg/mL; added directly to cell culture medium as a pretreatment; cells were pretreated with *C. longa* extract 1 h before salsolinol exposure; effects assessed at 24 h and 48 h after treatment.	Water extraction of C. longa extract was performed by boiling 100 g in 1.000 mL distilled water for 15 min over a low flame. The flask was subsequently plugged, removed from the heat, and allowed to cool. The extract was filtered and dried to prepare the required concentrations after cooling the content of the flask.	In vitro	Extract has a neuroprotective effect in SH-SY5Y cells and seemed to exhibit a clear dose-dependence when used in higher concentrations.	[11]
	Rhizome	Curcumin	USA	Standardized extract; 100 mg/kg; injected with sunflower seed oil as a vehicle, twice a day, for 50 days.	Standardized extract (commercial Sigma-Aldrich).	In vivo	The protective effects of curcumin against ROT-induced dopaminergic neuronal oxidative damage were mediated by activation of the Akt/Nrf2 signaling pathway.	[12]
Velvet bean (*Mucuna pruriens* L.)	Seeds	Natural Levodopa	USA	Seed powder; 12.5 g to 17.5 g per dose, equivalent to standard synthetic levodopa doses; orally.	Pulverization (dried and ground seeds), standardized.	In vivo	Dopaminergic replacement. Rapid onset of action, reduced dyskinesia compared to synthetic drugs	[25]
Wormwood (*Artemisia absinthium* L.)	Aerial parts	Artemisinin	Iran	Ethanolic extract; 25–200 µg/mL; single pretreat exposure following extract pretreatment.	A total of 200 g of the dried aerial parts was powdered, and the provided powder was percolated with 1500 mL of EtOH 70% for 72 h. After filtering the extract, the solvents were allowed to evaporate at 45 °C under reduced pressure to obtain the crude extracts.	In vitro	*A. absinthium* exerts its effect through inhibiting oxidative stress parameters, and it can be considered a promising candidate to be used in combination with the conventional medications for the treatment of neurodegenerative disorders.	[7]

### 2.3. Natural Compounds with Therapeutic Potential for Parkinson’s Disease

The growing body of experimental and clinical evidence highlights numerous natural compounds with therapeutic potential in Parkinson’s disease (PD).

A major category is represented by polyphenols and phenolic compounds, which are widely recognized for their antioxidant and cytoprotective properties. Curcumin, the principal polyphenol derived from *Curcuma longa*, targets oxidative stress, mitochondrial dysfunction, and apoptotic signaling. Experimental studies demonstrate that curcumin activates the Akt/Nrf2 pathway, enhances endogenous antioxidant defenses (GSH, SOD), preserves tyrosine hydroxylase (TH) expression, and attenuates dopaminergic neuronal loss in rotenone- and 6-OHDA-induced PD models [12,23]. Similarly, carnosic acid, a phenolic diterpene isolated from *Rosmarinus officinalis*, exerts neuroprotection by inhibiting lipid peroxidation, restoring glutathione metabolism, suppressing JNK and p38 MAPK activation, and modulating apoptotic regulators such as Bcl-2, Bax, and caspase-3 [18].

Another important group comprises flavonoids and flavonoid-rich extracts. Standardized safflower flavonoid extract (SAFE) from *Carthamus tinctorius* targets α-synuclein aggregation, oxidative stress, astrocyte activation, and dopaminergic dysfunction. SAFE treatment improves motor behavior, restores dopamine levels, upregulates TH and dopamine transporter expression, and partially normalizes extracellular space diffusion parameters in PD models [16]. *Ginkgo biloba* extracts (EGb and EGb761), which are rich in flavonoids and terpenoids, act on oxidative stress pathways, apoptotic signaling, and metal homeostasis. EGb enhances SOD and GSH-Px activity, reduces MDA levels, modulates Bcl-2/Bax balance, inhibits caspase-3 activation, and prevents copper dysregulation in MPP^+^-induced PD models [9,10].

Carotenoids and apocarotenoids are exemplified by crocin from *Crocus sativus* (saffron). Crocin and saffron extracts target oxidative stress, mitochondrial dysfunction, cholinergic imbalance, and dopaminergic neuronal survival. In *Drosophila* and murine PD models, saffron reduced ROS, restored GSH and thiol levels, improved mitochondrial enzyme activity, preserved TH-positive neurons, and normalized dopamine levels, translating into improved locomotor function and neuroprotection. Transcriptomic analyses indicate that saffron modulates multiple neuroprotective pathways, including apoptosis and inflammation [15].

A distinct class is formed by alkaloids and amino acid-related compounds, notably L-DOPA-containing plants. *Mucuna pruriens* seeds contain significant levels of natural L-DOPA alongside antioxidant and anti-inflammatory constituents. Clinical trials indicate that *M. pruriens* improves motor symptoms, shortens time to the “on” state, prolongs therapeutic benefits, and reduces adverse effects, including dyskinesia, compared with conventional levodopa formulations [25]. These effects suggest combined dopaminergic replacement and neuroprotective actions. Phenolic acids and related metabolites derived from spices also show therapeutic relevance. Cinnamon (*Cinnamomum verum*) is metabolized to sodium benzoate, which targets neuroinflammation, oxidative stress, and mitochondrial quality control. Sodium benzoate upregulates DJ-1 and Parkin, suppresses inducible nitric oxide synthase, and protects dopaminergic neurons in MPTP-induced PD models, resulting in improved motor function [26,27].

Quinones and terpenoid-related compounds include thymoquinone from *Nigella sativa*. Thymoquinone targets lipid peroxidation, ROS generation, and dopaminergic neuronal survival. In 6-OHDA-induced PD models, it reduces MDA levels, restores SOD activity, preserves substantia nigra neurons, and improves motor behavior [21]. Whole-seed extracts of *Nigella sativa* further demonstrate anti-cataleptic effects and normalization of neurochemical parameters in drug-induced Parkinsonism [20].

Finally, saponins and ginsenosides, particularly from *Gynostemma pentaphyllum* and *Panax ginseng*, represent an emerging therapeutic class. Gypenosides attenuate L-DOPA-induced dyskinesia by modulating ΔFosB expression and ERK1/2 phosphorylation without compromising anti-Parkinsonian efficacy [24]. Ginseng water extract targets α-synuclein aggregation, dopaminergic neuronal survival, and gut microbiota dysregulation, highlighting a gut–brain axis mechanism that is relevant to PD pathology [29].

## 3. Alzheimer’s Disease

### 3.1. Oxidative Stress and Neuronal Damage in Alzheimer’s Treatment

The neuropathological features of Alzheimer’s disease include extracellular amyloid-β plaque deposition, intracellular hyperphosphorylation of the tau gene, chronic neuroinflammation, synaptic dysfunction, oxidative stress, mitochondrial damage, and widespread neuronal loss that underlies cognitive decline [30].

Experimental evidence consistently demonstrates that oxidative stress is both a cause and consequence of Aβ-induced neurotoxicity. In animal models of AD, increased levels of malondialdehyde (MDA), protein carbonyls, and nitric oxide (NO), along with altered antioxidant enzyme activities, reflect severe redox imbalance.

Inhalation of *Juniperus communis* volatile oil in an amyloid β(1–42)-induced rat model significantly reduced hippocampal acetylcholinesterase (AChE) activity and attenuated oxidative stress markers, including MDA and protein carbonyl levels, while restoring antioxidant enzyme activities such as superoxide dismutase (SOD) and catalase [31]. These findings suggest that reducing the oxidative burden may contribute to preserving cholinergic function and mitigating neuronal damage associated with AD.

Similarly, *Lavandula angustifolia* essential oil demonstrated strong antioxidant and neuroprotective effects in both in vivo and in vitro models. In scopolamine-induced cognitive impairment in mice, lavender oil significantly increased SOD and glutathione peroxidase (GPx) activities and reduced AChE and MDA levels. In PC12 cells exposed to H_2_O_2_, the oil decreased intracellular ROS accumulation, nitric oxide release, and mitochondrial membrane potential loss, thereby preventing oxidative cytotoxicity [32]. These results emphasize the relevance of antioxidant modulation in counteracting oxidative neuronal injury.

The brain is particularly vulnerable to oxidative injury due to its high oxygen consumption, abundance of polyunsaturated fatty acids, and relatively limited antioxidant capacity. In AD, Aβ peptides—particularly Aβ1–42 oligomers—induce excessive ROS generation, mitochondrial dysfunction, and lipid peroxidation, thereby initiating apoptotic signaling cascades. Studies using neuronal cell lines and animal models consistently demonstrate that oxidative stress precedes and amplifies synaptic loss and cognitive impairment. At the cellular level, Aβ-induced oxidative stress disrupts mitochondrial function, activates intrinsic apoptotic pathways, and amplifies inflammatory signaling cascades. Experimental models employing hippocampal injection of Aβ fragments (Aβ25–35 or Aβ1–42) consistently demonstrate increased ROS generation, reduced antioxidant enzyme activity, mitochondrial cytochrome c release, caspase activation, and neuronal apoptosis [33]. These findings reinforce the concept that oxidative stress operates both upstream and downstream of Aβ accumulation, creating a vicious cycle that perpetuates neuronal damage.

#### 3.1.1. Interaction Between Oxidative Stress and Cholinergic Dysfunction

Cholinergic deficits are a hallmark of AD pathology, and oxidative stress is closely intertwined with cholinergic neuron degeneration. Excessive ROS can impair acetylcholine synthesis, accelerate acetylcholinesterase activity, and compromise synaptic transmission. Several plant-derived compounds reviewed here exert dual effects by reducing oxidative stress while modulating cholinergic signaling.

*Murraya koenigii* leaf extract significantly enhanced endogenous antioxidant defenses in aged mice by increasing levels of glutathione peroxidase, superoxide dismutase, catalase, and reduced glutathione, while simultaneously decreasing lipid peroxidation and nitric oxide levels. Importantly, the extract reduced cholinesterase activity, thereby increasing acetylcholine availability in the brain [34]. This dual antioxidant–cholinergic mechanism suggests a synergistic neuroprotective effect that is relevant to AD pathology.

Comparable outcomes were observed with *Coriandrum sativum*, whose extracts and volatile oil demonstrated antioxidant, anti-inflammatory, and neuroprotective properties. In Aβ(1–42)-treated rat models, inhalation of coriander volatile oil improved spatial memory performance, reduced oxidative stress markers such as MDA, normalized GPx activity, and prevented DNA fragmentation in hippocampal tissue [35]. Further studies showed that coriander volatile oil also exerted anxiolytic and antidepressant-like effects, while decreasing catalase activity and increasing glutathione levels, highlighting its multifaceted role in counteracting oxidative and behavioral disturbances in AD [36]. Research suggests that *Coriandrum sativum* extracts and essential oils may modulate cholinergic signaling related to cognitive function by inhibiting acetylcholinesterase (AChE) activity and improving behavior in experimental models [37].

Oxidative stress is closely associated with cholinergic dysfunction, a core neurochemical deficit in AD. Several studies [15,38] demonstrate that plant extracts with antioxidant properties also inhibit cholinesterase activity, thereby enhancing cholinergic neurotransmission while reducing the oxidative burden. At the cellular level, oxidative stress contributes directly to Aβ-induced neuronal apoptosis. Studies on *Salvia officinalis* and its active compound rosmarinic acid provide compelling evidence of antioxidant-mediated neuroprotection. In PC12 cells exposed to Aβ(1–42), rosmarinic acid significantly reduced ROS formation, lipid peroxidation, DNA fragmentation, caspase-3 activation, and tau hyperphosphorylation, while inhibiting p38 MAP kinase activation [39]. These findings suggest that antioxidant compounds can interrupt multiple downstream pathways of oxidative and amyloid-induced toxicity.

The relevance of these mechanisms extends to clinical settings. A double-blind, randomized, placebo-controlled trial demonstrated that *Salvia officinalis* extract significantly improved cognitive function in patients with mild-to-moderate AD, with a favorable safety profile [40]. Research shows that extracts from *Salvia officinalis* can enhance cognitive function and inhibit acetylcholinesterase activity, indicating modulation of cholinergic signaling, which is important to cognitive function [41]. Similarly, *Melissa officinalis* extract improved cognitive outcomes and reduced agitation in AD patients, effects that may be partially attributed to its antioxidant and neuroprotective properties [42,43].

#### 3.1.2. Oxidative Stress, Apoptosis, and Intracellular Signaling Pathways

Beyond direct radical scavenging, several phytochemicals exert neuroprotective effects by modulating intracellular signaling pathways that govern oxidative stress responses and apoptosis. The Akt/GSK-3β pathway is particularly relevant in AD, as GSK-3β activation contributes to tau hyperphosphorylation and neuronal apoptosis.

In PC12 cells exposed to Aβ1–42, 6-gingerol significantly increased phosphorylated Akt (p-Akt) and phosphorylated GSK-3β (p-GSK-3β), thereby inhibiting pro-apoptotic signaling and enhancing cell survival [44]. These findings suggest that oxidative stress reduction and kinase modulation are mechanistically interconnected. Similarly, curcumin derivatives such as FMeC1 not only reduced the Aβ burden but also suppressed glial activation and cognitive deficits in APP/PS1 mice, indicating that attenuation of oxidative stress-related signaling cascades contributes to disease modification [45].

Garlic-derived compounds further illustrate this mechanism. Chauhan and Sandoval [46] showed that AGE preserved approximately 80% of neuronal cells from ROS-mediated injury when administered as a pretreatment, suggesting a neuropreservation effect that extends beyond acute antioxidant activity. This supports the notion that strengthening endogenous defense systems can increase neuronal resilience against chronic oxidative insults characteristic of AD.

Notably, the combination of EGb 761 and HBO exerted synergistic effects, suggesting that enhancement of oxygen availability together with antioxidant and anti-apoptotic mechanisms can more effectively counteract oxidative neuronal injury [33,47]. At the signaling level, these interventions were associated with modulation of the NF-κB pathway, which serves as a critical intersection between oxidative stress, inflammation, and cell survival [47].

The APOE4 gene variant, the strongest genetic risk factor for late-onset Alzheimer’s disease, has been associated with increased oxidative stress, impaired lipid metabolism, and enhanced neuronal vulnerability. The APOE4 gene variant is the single greatest genetic risk factor for developing late-onset Alzheimer’s disease [48]. Carrying one copy increases risk by 3–4 times, and two copies can raise it by 12–15 times. While directly targeting APOE4 with drugs remains a challenge, promising research focuses on mitigating its damaging effects. Certain natural compounds show potential in preclinical studies to protect brain cells from the specific toxic pathways—like amyloid buildup, tau tangles, and neuroinflammation—that APOE4 accelerates. Recent studies continue to illuminate APOE4’s complex mechanisms, from altering brain network connectivity influenced by diet (e.g., coffee/tea) [49] to disrupting cellular energy and increasing seizure susceptibility, paving the way for novel therapeutic strategies [50].

### 3.2. Phytochemical-Based Therapeutic Strategy for Alzheimer’s Treatment

Given the limited efficacy and side-effect profile of currently approved pharmacological agents—which are largely restricted to acetylcholinesterase inhibitors and NMDA receptor antagonists—there is increasing scientific interest in phytochemical-based therapeutic strategies. Plant-derived bioactive compounds (summarized for 23 plants) offer a pleiotropic mode of action, simultaneously targeting multiple pathogenic pathways involved in AD (Table 2).

#### 3.2.1. Modulation of Amyloidogenesis and Tau Pathology by Phytochemicals

A central therapeutic target in AD is the reduction in Aβ production, aggregation, and toxicity. Several plant-derived compounds reviewed here demonstrate the ability to interfere directly with amyloidogenic processing or downstream amyloid toxicity. Flavonoid-rich extracts of *Capparis spinosa* significantly downregulated the expression of genes that are critically involved in Aβ generation, including BACE-1, APP, PSEN-1, and PSEN-2, in an Aβ-injected rat model [51]. Given that BACE-1 and γ-secretase components (PSEN-1 and PSEN-2) are essential for Aβ formation, these findings suggest that high concentrations of rutin and quercetin in *C. spinosa* may exert disease-modifying effects by suppressing amyloidogenic pathways at the transcriptional level.

#### 3.2.2. Cholinergic Enhancement and Synaptic Protection

Cholinergic dysfunction remains one of the most clinically relevant features of AD, and several phytochemicals reviewed here exhibit strong anticholinesterase activity. *Syzygium aromaticum* (clove) and its principal constituent eugenol showed potent inhibition of acetylcholinesterase and butyrylcholinesterase, with kinetic analyses revealing mixed and non-competitive modes of inhibition, respectively [52]. These findings suggest that clove-derived compounds may sustain acetylcholine availability through mechanisms distinct from conventional cholinesterase inhibitors.

Similarly, *Portulaca oleracea* demonstrated strong acetylcholinesterase inhibition supported by molecular docking and molecular dynamics simulations, indicating stable ligand–enzyme interactions [55]. The identification of dopamine and norepinephrine as potential bioactive constituents further suggests that phytochemicals may modulate multiple neurotransmitter systems beyond the cholinergic axis.

Synaptic preservation is another critical therapeutic target addressed by phytochemical interventions. *Borago officinalis* extract prevented Aβ-induced disruption of long-term potentiation in the hippocampal dentate gyrus and preserved total thiol content, indicating protection of synaptic plasticity through antioxidant mechanisms [58]. Behavioral studies confirmed that borage administration attenuated memory impairment and restored hippocampal antioxidant capacity [82]. In transgenic APP/PS1 mice, L-3-*n*-butylphthalide derived from *Apium graveolens* significantly increased synapse density, dendritic spine number, and synaptic protein expression, while reducing Aβ plaques and neuroinflammation, effects that were mediated via the Wnt/β-catenin signaling pathway.

Aged garlic extract (AGE) has been examined as a neuroprotective intervention with both antioxidant and amyloid-modulating properties. In Aβ-induced cognitive impairment paradigms, oral AGE improved working memory and ameliorated cholinergic neuronal loss, while also modulating glutamatergic and GABAergic markers (e.g., increased VGLUT1 and GAD) in hippocampus [70]. In PC12-based neurotoxicity assays, specific solvent fractions of aged garlic demonstrated antioxidant capacity, reduced intracellular ROS, protected against Aβ-mediated LDH release and cytotoxicity, and attenuated learning and memory deficits in mouse behavioral tests [71].

Mechanistic work further implicates S-allyl-L-cysteine (SAC) as an active contributor: AGE and SAC protected neuronal cultures from ROS insults and preserved synaptic proteins such as SNAP25; in APP–transgenic mice, AGE/SAC increased synaptic protein levels, which are typically reduced in AD models [72]. In Tg2576 mice, AGE ameliorated early cognitive deficits [46], and dietary garlic reduced Aβ40/Aβ42 while increasing sAPPα, consistent with shifting APP processing away from amyloidogenic pathways [83]. Across these studies, garlic-based preparations converge on synaptic preservation, oxidative injury mitigation, and Aβ lowering—three interdependent dimensions of disease progression.Polyphenols and Flavonoids. *Capparis spinosa* leaves and fruits are particularly rich in flavonoids, especially rutin and quercetin, as quantified by HPLC analysis. In an Aβ-injected rat model, flavonoid-rich extracts significantly downregulated the gene expressions of BACE-1, APP, PSEN-1, and PSEN-2, all of which are critical components of amyloidogenic APP processing. These findings suggest that *Capparis spinosa* exerts disease-modifying potential by targeting transcriptional regulation of amyloid-related and inflammatory genes, supporting its use as a dietary supplement for AD risk reduction [51]. Borage extract demonstrated protective effects against Aβ(25–35)-induced synaptic dysfunction by restoring long-term potentiation (LTP) parameters in the dentate gyrus and preserving hippocampal thiol content [58]. Behavioral studies further showed improvements in spatial and avoidance memory, accompanied by restoration of antioxidant capacity (FRAP) in hippocampal tissue [82]. These results position *Borago officinalis* as a modulator of synaptic plasticity and redox homeostasis. Coriander leaf extracts suppressed Aβ42-induced ROS generation, glial proliferation, and ERK activation in both cellular and *Drosophila* AD models, improving survival without altering Aβ expression levels (*C. sativum* study). In vivo studies using inhaled coriander volatile oil demonstrated improvements in spatial memory, attenuation of oxidative stress markers, and antiapoptotic effects in Aβ(1–42)-treated rats [35,36]. Additionally, dietary coriander leaves enhanced memory performance and reversed scopolamine- and aging-induced amnesia, likely through antioxidant and hypolipidemic mechanisms [84].

Alkaloids and Nitrogen-Containing Compounds. Total alkaloidal extracts from *Murraya koenigii* significantly enhanced endogenous antioxidant enzymes (SOD, catalase, GPx, GR) and reduced lipid peroxidation and nitric oxide levels in aged mice [34]. Concomitantly, cholinesterase activity was reduced, leading to increased acetylcholine availability. These dual antioxidant and cholinergic effects support the relevance of curry leaf alkaloids in dementia-related conditions. Multiple studies indicate that *Berberis vulgaris* extracts and their alkaloid constituents exert potent antioxidant and anticholinesterase effects. Berberine and related isoquinoline alkaloids significantly inhibited AChE and BuChE activities and reduced TBARS, NO, and DPPH oxidation, implicating them in protection against oxidative and cholinergic dysfunction in AD [57,85]. One specific alkaloid demonstrated strong inhibition of human BuChE (IC_50_ = 0.82 μM), supported by molecular docking studies [56]. Piperine and standardized *Piper nigrum* extracts improved memory performance, reduced cholinesterase activity, attenuated lipid peroxidation, and prevented neurodegeneration in aluminum chloride and AF64A-induced AD models [63]. Additional studies demonstrated inhibition of amyloid plaque formation and tau oligomerization, highlighting black pepper as a preventive dietary agent against AD progression [1].

Essential Oils and Terpenoids. Eugenol, the main constituent of clove essential oil, exhibited strong inhibitory activity against both AChE and BuChE, with mixed-type inhibition for AChE and non-competitive inhibition for BuChE [52]. These findings provide a mechanistic basis for the traditional use of clove in cognitive disorders. Lavender oil improved scopolamine-induced cognitive deficits in mice and protected PC12 cells from H_2_O_2_-induced cytotoxicity by reducing ROS, NO, LDH release and mitochondrial membrane potential loss, while enhancing antioxidant enzyme activities [32]. Inhalation of juniper volatile oil significantly reduced hippocampal AChE activity and restored antioxidant balance in Aβ(1–42)-treated rats, supporting its role in mitigating oxidative stress-related neurotoxicity [31].

Multi-Target Phytochemical Extracts and Novel Compounds. L-3-*n*-Butylphthalide (L-NBP) treatment reduced amyloid plaque burden, neuroinflammation, and synaptic loss while enhancing synapse-associated proteins in aged APP/PS1 mice. Restoration of synaptic function was linked to modulation of the Wnt/β-catenin pathway [86]. Fenugreek seed powder attenuated AlCl_3_-induced cognitive deficits, oxidative stress, inflammation, amyloid burden, and tau pathology by regulating Akt/GSK3β signaling and suppressing proinflammatory mediators [60].

Clinical Evidence-Based Herbal Agents. Rosmarinic acid protected PC12 cells from Aβ-induced toxicity by inhibiting ROS formation, lipid peroxidation, DNA fragmentation, caspase-3 activation, and tau hyperphosphorylation [39]. Importantly, *Salvia officinalis* extract demonstrated significant cognitive benefits in randomized, placebo-controlled clinical trials in patients with mild-to-moderate AD [40].

Phenylpropanoids and aromatic aldehydes. Cinnamon-derived phytochemicals prominently target amyloidogenic processing and protein aggregation. In APP-expressing CHO cells, phenylpropanoids such as medioresinol and cryptamygin A significantly reduced Aβ40 production by downregulating β-secretase levels and sAPPβ formation, directly implicating them in upstream modulation of APP processing [68]. This positions these compounds as anti-amyloidogenic agents acting at the level of Aβ generation. At the aggregation level, orally administered cinnamon extract (CEppt) inhibited the formation of toxic Aβ oligomers, reduced 56 kDa oligomeric species, and improved cognitive performance in fly and transgenic mouse models of AD [87]. Complementarily, aqueous extracts of *Ceylon cinnamon* inhibited tau aggregation and promoted disassembly of preformed tau filaments, with A-linked proanthocyanidins and cinnamaldehyde being identified as major contributors [88]. These data suggest that cinnamon compounds uniquely bridge amyloid and tau pathologies.

Cinnamon metabolites also target neuroinflammation and oxidative stress. Sodium benzoate suppressed microglial ROS production induced by LPS and Aβ, inhibited activation of p21rac signaling, reduced oxidative damage in the hippocampus, and attenuated the Aβ burden and cognitive decline in 5XFAD mice [69]. Thus, cinnamon-derived compounds act on β-secretase activity, Aβ oligomerization, tau aggregation, microglial redox signaling, and cognitive function.

Organosulfur compounds and aged extracts. Garlic-derived compounds, particularly in aged preparations, exhibit strong antioxidant, synaptoprotective, and amyloid-lowering properties. Aged garlic extract (AGE) improved working memory in Aβ-injected rats and mitigated loss of cholinergic neurons while modulating glutamatergic (VGLUT1) and GABAergic (GAD) markers in the hippocampus [70]. In vitro, AGE fractions reduced ROS accumulation, protected PC12 cells from Aβ-induced cytotoxicity, and attenuated memory deficits in mouse behavioral assays [71]. At the molecular level, S-allyl-L-cysteine (SAC) has been identified as a key bioactive component mediating AGE’s effects. AGE and SAC protected neurons from ROS-induced damage, preserved presynaptic proteins such as SNAP25 and synaptophysin, and exerted neurorescue effects in APP–transgenic mouse models [46,72]. Importantly, dietary garlic shifted APP processing toward the non-amyloidogenic pathway by increasing sAPPα and reducing Aβ40 and Aβ42 levels in Tg2576 mice [83]. Therefore, garlic compounds target oxidative stress, synaptic integrity, cholinergic dysfunction, and amyloid burden.

Carotenoids and apocarotenoids. Saffron-derived carotenoids uniquely emphasize Aβ clearance, BBB integrity, and clinical translatability. Trans-crocetin enhanced degradation of Aβ42 in monocytes derived from AD patients by upregulating cathepsin B, directly implicating lysosomal clearance mechanisms in human immune cells [73]. In vivo, saffron extract tightened BBB function, enhanced Aβ transport and clearance, reduced amyloid load, upregulated synaptic proteins, and suppressed neuroinflammation in 5XFAD mice [74]. Clinically, *Salvia officinalis* extract demonstrated comparable efficacy to donepezil in mild-to-moderate AD and to memantine in moderate-to-severe AD, with favorable safety profiles [40]. Collectively, saffron compounds target Aβ clearance, BBB dysfunction, neuroinflammation, cholinergic enzymes, and cognitive decline.

Curcuminoids and optimized turmeric extracts. Curcumin and its derivatives constitute one of the most extensively characterized phytochemical classes in AD research. Curcumin suppresses AChE activity and gene expression, normalizes oxidative stress markers, and improves memory in toxin- and STZ-induced dementia models [76,89]. Biophysical studies demonstrate that curcumin directly binds Aβ oligomers and fibrils and disrupts β-sheet formation by interacting with the KLVFF core motif [90,91]. Optimized formulations, such as the curcumin derivative FMeC1 and the standardized extract HSS-888, exhibit superior efficacy in reducing insoluble Aβ, phosphorylated tau, and microglial inflammation compared with native curcumin [45]. Dietary curcumin also reversed Aβ-induced synaptic loss and cognitive deficits in aged rats [92]. Thus, curcuminoids target Aβ aggregation, tau phosphorylation, cholinergic dysfunction, oxidative stress, neuroinflammation, and synaptic loss.

Sesquiterpene Lactones. Dihydroartemisinin (DHA) significantly improved learning and memory in APP/PS1 mice by promoting autophagosome–lysosome fusion and restoring autophagic flux, leading to enhanced clearance of Aβ fibrils and reduced neuronal apoptosis [79]. These effects were accompanied by increased LC3II/I ratios and decreased p62 accumulation, even under pharmacological inhibition of autophagosome–lysosome fusion [81]. Artemisinin B demonstrated potent anti-neuroinflammatory activity by suppressing TLR4–MyD88–NF-κB signaling, reducing microglial release of IL-1β, IL-6, and TNF-α, and improving spatial memory in AD mice [80]. Similarly, artemisinin improved cognitive function in 3xTg AD mice by reducing Aβ and tau deposition and activating the ERK/CREB pathway, thereby enhancing neuronal survival both in vivo and in SH-SY5Y cells [79].

Lignans and Metabolic Modulators. Network pharmacology and experimental validation identified *Schisandra chinensis* Fructus SCF lignans, including gomisin A and schisandrin, as modulators of key AD-related targets such as PTGS2 (COX-2) and acetylcholinesterase (AChE) [93]. In Aβ25–35-infused rats, dietary SCF reduced hippocampal Aβ deposition, suppressed neuroinflammation, improved memory performance, and normalized glucose and lipid metabolism [93]. Preservation of neurotrophic factors such as BDNF and CNTF further supports its neuroprotective profile.

Multifunctional Antioxidant Phytochemicals. *O. baccatus* leaf extract exhibited strong antioxidant capacity, significant AChE inhibition, and marked reduction in Aβ aggregation in vitro. In vivo, it improved spatial and working memory and restored hippocampal neuronal density in scopolamine-induced mice, highlighting its multifunctional anti-Alzheimer’s potential [94].

Comparative antioxidant studies further demonstrated that *Ginkgo biloba* exhibits moderate radical-scavenging activity relative to Yerba Mate and green tea, underscoring the heterogeneity of antioxidant efficacy among plant-derived interventions with relevance to AD [95].

## 4. Conclusions

Within all of these phytochemical classes being presented, there is an overarching theme of multi-target engagement that is consistent with the pathophysiological feature of PD and AD. Ginger and garlic reduce oxidative stress and inflammatory signaling and support cholinergic and synaptic function. Cinnamon- and curcumin-based strategies extend to proteinopathy modulation, influencing Aβ production/assembly and tau aggregation. Saffron shows the strongest translational potential to clinical settings, and mechanistic data supports improved clearance and BBB effects. However, interpretation must still be refined to account for the model constraints, variable extracts, the dosing and formulation variability, and the significant discontinuity between preclinical efficacy and clinical disease modification. Taken in aggregate, the data lend credibility to the rationale of phytochemical-based therapeutics as adjunctive or preventive targets, and—when optimizing targeting improves activity (e.g., curcumin derivatives)—as an indication to further develop pipelines.

The collective evidence reviewed here underscores oxidative stress as a pivotal driver of neuronal damage in Parkinson’s disease and Alzheimer’s disease. While most data remain preclinical, the convergence of mechanistic findings strongly supports further clinical investigation of antioxidant-based adjunct therapies aimed at slowing neurodegeneration and improving outcomes in PD. By reducing ROS generation, enhancing endogenous antioxidant defenses, stabilizing mitochondrial function, and attenuating apoptosis, interventions such as *Ginkgo biloba* extract (EGb 761) exert multifaceted neuroprotective effects. Plant-derived compounds and extracts consistently demonstrate the capacity to attenuate oxidative injury, stabilize mitochondrial function, regulate apoptotic pathways, and preserve dopaminergic neurons across diverse experimental models.

## Figures and Tables

**Table 2:** Positive pharmacological activity of bioactive plant compounds on Alzheimer’s disease.

Herb	Physiological Part Used	Major Component	Country	Type of Extract/Concentration	Method of Obtainment	Type of Study	Mechanism of Action	References
Caper (*Capparis spinosa*)	Leaf, fruit	Rutin, quercetin	Iran	Hydroalcoholic extracts (20 mg/kg/BW); orally, once daily for 6 weeks.	The flavonoid-rich extract of Capparis spinosa was made by hydroalcoholic extraction of dried, powdered plant material with an ethanol–water mixture. The solvent was then evaporated to produce a dry extract for experimental use.	In vivo	*C. spinosa* has the potential to downregulate inflammation-involved genes in AD.	[51]
Clove (*Syzygium aromaticum* L.)	Dried flower buds	Eugenol	India	Hydroalcoholic extract (10–1000 µm/mL), clove oil (49.7 µm/mL), eugenol (49.7 µm/mL); directly added into the vitro assay system; single exposure per assay.	The plant extract: Cold-macerated with a hydroalcoholic solvent (25 g powder, 72 h of shaking). The extract was filtered, pooled, evaporated under low pressure, and then lyophilized. Yield is 8% *w*/*w*. Clove oil: The powdered clove (10 g) was cooked in water at 100 °C for 5 h. The oil was separated, dried with anhydrous sodium sulfate, and kept at 4 °C. Yield of 6% *v*/*w*.	In vitro	Eugenol demonstrated highest acetylcholinesterase inhibitory ability among the extract and oil *S. aromaticum* extract and its oil, along with eugenol, have potential anti-cholinesterase properties.	[52]
Common lavender (*Lavandula angustifolia*)	Herbs	Linalool, linalyl acetate	China	Essential oil (12 µg/mL; 100 mg/kg); intraperitoneal, once daily, 10 days.	Standardized extract (commercial source—Enhui, Shanghai, China).	In vivo/vitro	Improves the scopolamine-induced cognitive deficit in mice and protects rat pheochromocytoma cells against H_2_O_2_-induced cytotoxicity. The neuroprotective effect is mainly related to the modification of cholinergic neuronal systems and the modulation of oxidative stress.	[32]
Bell pepper (*Capsicum annuum*)	Fruits	Capsacinoids	Korea	Ethanolic extract (3 g/day); orally; 28 days.		In vivo	Prevents the memory deficit and exacerbation of insulin resistance by blocking tau phosphorylation and β-amyloid accumulation in diabetic rats with experimentally induced Alzheimer’s-like dementia.	[53]
Curry leaf (*Murraya koenigii*)	Leaves	Girinimbine, mahanimbine, murrayanine	Malaysia	Alkaloidal extract (10, 20, and 40 mg/kg); orally, once daily/15 days	To extract *Murraya koenigii* leaf alkaloids, shade-dried leaves were powdered and extracted with 95% methanol using Soxhlet method. The extract was concentrated, acidified (0.5 M H_2_SO_4_), washed with chloroform, basified (pH 10, ammonia), re-extracted with chloroform, and evaporated to obtain the total alkaloid fraction (0.124% *w*/*w*).	In vivo	Decreased lipid peroxidation, as well as nitric oxide levels, and enhanced brain antioxidants such as glutathione peroxidase; reduced glutathione, glutathione reductase, superoxide dismutase, and catalase and significantly prevented the brain from aging-induced oxidative stress.	[34]
Licorice (*Glycyrrhiza glabra*)	Herbs	licorice	Japan	Yokukansan (7.5 g/day, containing 4.5 g of licorice); oral, chronic use prior to hospital admission.	Standardized extract.	In vivo (human case)	Its therapeutic effect is attributed to the modulation of neuronal activity, particularly by influencing neurotransmitter systems involved in behavior and agitation.	[54]
Purslane (*Portulaca oleracea*)	Stem	Dopamine and norepinephrine	USA	Alkaloidal extract (100 µg/mL); added directly to the reaction mixture; single exposure per assay.	Plant samples weighing 120 g were ground into a powder. After that, each sample was treated with 95% ethanol and refluxed for two hours in order to extract the alcohol. After additional evaporation and air drying, the extract was suspended in deionized water. The suspension’s pH was brought down to 2.0. After overnight incubation, the acidic aqueous solution from the alkaloidal extracts was filtered, the pH was adjusted to 10.0, and the extract was extracted using chloroform. After evaporating, the chloroform layers were allowed to air dry.	In vitro	The extract exhibited 74.2% inhibition against acetylcholinesterase.	[55]
Common juniper (*Juniperus communis*)	Berry	a-thujene, a-pinene, sabinene, myrcene, limonene, c-terpinene, terpinen-4-ol, followed by lower quantities of b-elemene, b-caryophyllene, germacrene D, eremophylene.	Romania	Volatile oil (200 μL); inhalation, once daily/60 min period/21 continuous days.	The juniper berry volatile oil was produced by hydro-distillation for 2 h.	In vivo	Juniper volatile oil presents both antiacetylcholinesterase and antioxidant activities and may contribute to increasing the levels of acetylcholine in cholinergic neurons, while simultaneously helping to prevent further degradations caused by radical oxygen species.	[31]
Barberry (*Berberis vulgaris*)	Roots bark	Bersavine, muraricine, and berbostrejdine	Austria	Alkaloidal extract (IC50 = 0.82 ± 0.10 Μm); added directly to the enzyme reaction mixtures; single exposure per assay.	The root bark of B. vulgaris (30 kg) was dried, chopped, extracted with 95% ethanol (EtOH), denaturated with methanol (MeOH) three times (3 × 30 min) under a reflux, and filtered. The filtrate was evaporated under a vacuum to obtain an EtOH extract, which was suspended in 2% HCl (6 L), diluted with distilled H_2_O (6 L), and filtered.	In vitro	It acts predominantly as an inhibitor of cholinesterases (particularly BuChE), blocking the degradation of acetylcholine.	[56]
	Roots	Berberine	Egypt	Barberry crude extract; ethanolic extract (0.2–1.0 mg/mL); added directly to the vitro test systems; single exposure per assay.	The dried powdery roots were exhaustively defatted with petroleum ether and then dried in fresh air to evaporate the solvent. The dried roots were used to prepare the ethanolic crude extract by subjecting to steam distillation method using Soxhlet apparatus, in which the powder was added in glass thimble and boiled ethanol extracted the active compounds for 8 h.	In vitro	The barberry crude extract and its active alkaloid, berberine, suppress lipid peroxidation, suggesting a promising use in the treatment of hepatic oxidative stress, Alzheimer, and idiopathic male factor infertility.	[57]
Borage (*Borago Officinalis* L.)	Leaves	-	Iran	Hydroalcoholic extract (100 mg/kg); intraperitoneal, once daily/7 days	The powdered material was soaked into aqueous ethanol (80%) for 1 week with occasional shaking. The extract was filtered through a Whatman filter paper and evaporated to dryness under reduced pressure at a maximum of 40 °C using a rotary evaporator.	In vivo	*Borago officinalis* (borage) extract on amyloid β (Aβ) induced long-term potentiation disruption in hippocampal dentate gyrus.	[58]
Celery (*Apium graveolens*)	Seeds	L-3-*n*-butylphthalide (L-NBP)	Chine	Oral gavage, once daily/90 days 15 mg/kg.	L-NBP (purity > 98.5%) diluted in vegetable oil at a concentration of 3 mg/mL.	In vivo	L-NBP treatment significantly increased the number of synapses and apical dendritic thorns and the thickness of PSD and increased the expression levels of synapse-associated proteins.	[59]
Fenugreek (*Trigonella foenum-graecum*)	Seeds	Flavonoids	India	Seed powder mixed with 5% feed (2.5%, 5%, and 10%); oral, once a day/60 days.	Seeds germinated, powdered, and mixed into diet.	In vivo	Reported outcomes include amelioration of cognitive impairments, reduction in oxidative stress and inflammation, and modification of tau/amyloid-related protein patterns, suggesting multi-pathway neuroprotective effects.	[60]
Sichuan pepper (*Zanthoxylum bungeanum*)	Herbs	*N*-[2-(3,4-dimethoxyphenyl)ethyl]- 3-phenyl-acrylamide	China	*N*-[2-(3,4-dimethoxyphenyl)ethyl]-3-phenyl-acrylamide (gx-50); oral, once days/30 days.	Extracted/synthesized derivative from *Z. bungeanum*.	In vivo/vitro	gx-50’s effects include anti-amyloid oligomerization, anti-apoptotic action, and calcium toxicity reduction, indicating multiple neuroprotective pathways.	[61]
Black seed (*Nigella sativa*)	Seeds	Thymoquin one (TQ)	Japan	Thymoquinone solution; directly added to the cell culture medium; single pretreatment	Thymoquinone solution (10 mM) was prepared immediately before the experiment by dissolving 1.642 mg of TQ in a 1 mL of solution made of DMSO and culture medium. Final concentrations of TQ were prepared in the culture medium. Aβ1–42 was administered to cell cultures with or without TQ on day 13 in vitro for 72 h.	In vitro	Synaptic function was partly restored (vesicle recycling and firing), and Aβ aggregation in vitro was also inhibited.	[62]
Black pepper (*Piper nigrum*)	Seeds	Piperina	India	Standardized ethanolic extract; oral, once daily/15 days.	The seeds were powdered manually, and extraction was performed by using Soxhlet apparatus. The procedure took two days. The extract was filtered, vacuum-dried, and stored in a refrigerator until further use. The yield was 10.4%.	In vivo	Antioxidant activity, anti-inflammatory effects, inhibition of acetylcholinesterase, reduction in oxidative stress and neurodegeneration, and neuroprotective effects, improving cognitive function.	[63]
Three-lobed sage (*Salvia triloba*) *and* Black pepper (*Piper nigrum*)	Herbs/seeds	Salvia: hydroxybenzoic acid derivatives, caffeic acid derivatives (e.g., rosmarinic acid), ferulic acid as well as flavonoid derivatives; luteolin and quercetin. *Piper nigrum*: piperine	Egypt	Methanolic extract (mg/kg body weight); oral, once daily/3 months.	The aerial part of *Salvia triloba* L. and seeds of *Piper nigrum* were macerated in 500 mL of 70% methanol, left at room temperature for three days, and then filtered. The residue was repeatedly extracted with fresh methanol. The combined filtrates were evaporated under reduced pressure at 45 °C in a rotatory evaporator.	In vivo	Treatment of AD-induced rats with rivastigmine (reference drug), *Salvia triloba*, and *Piper nigrum*; total extracts significantly reduced the oxidative stress status and ameliorated the neurodegeneration characteristics of Alzheimer’s disease in rats.	[64]
Coriander (*Coriandrum sativum* L.)	Leaves	Rutin	South Korea	Ethanolic extract (200 μg/mL); added directly to the culture media; single exposure per assay.	C. sativum leaves were extracted twice in 30% ethanol at 100 °C with a reflux condenser for 3 h. The extract was filtered with a 50 μm filter and concentrated with a lyophilizer at −60 °C. The final yield was approximately 6.5 g of dried material (average yield, 6.5%). The dried material was mixed with fly food at the indicated concentrations or stored at −80 °C until further use.	In vitro	Suppression of Aβ42-induced ROS production; inhibition of glial cell proliferation; downregulation of ERK signaling pathway; antioxidant and neuroprotective effects.	[65]
	Fruits	Linalool, α-pinene, γ-terpinene, geranyl acetate, camphor and geraniol	Romania	Volatile oil (1%; 3%); inhalation, once daily/21 days.	Air-dried fruits of *C. sativum* var. microcarpum samples were subjected to hydro-distillation for 3 h using a Clevenger-type apparatus to obtain the volatile oil. The essential oil was dried over anhydrous sodium sulfate and kept at −4 °C.	In vivo	Volatile coriander oil is crucial for the development of spatial memory, particularly working and reference memories.	[35]
Coriander (*Coriandrum sativum* var. *microcarpum*)	Mature fruits	linalool, α-pinene, γ-terpinene, geranyl acetate, camphor and geraniol	Romania	Volatile oil (exact µL/L air depends on protocol); inhalation, once daily/21 days.	Air-dried fruits of coriander sample were subjected to hydro-distillation for 3 h using a Clevenger-type apparatus to obtain the volatile oil. The volatile oil was dried over anhydrous sodium sulfate and kept at −4 °C until analysis.	In vivo	Reduction in oxidative stress (↓ lipid peroxidation, ↑ antioxidant status); anxiolytic- and antidepressant-like behavioral effects; meuroprotection against Aβ1–42-induced oxidative damage.	[36]
Sage (*Salvia. officinalis* L.)	Aerial parts	Rosmarinic acid	Italy	A hydroalcoholic dry extract standardized to contain 9.9% rosmarinic (0.01–100 g/mL); directly to the culture medium; single administration.	Solvent extraction of sage leaves (ethanol/methanol); isolation/purification of rosmarinic acid.	In vitro	Prevents apoptosis and cell death; Polyphenolic action has a neuroprotective effect.	[39]
	Leaves	Rosmarinic acid	Iran	Alcoholic extract (60 drops/day); oral, for 4 months in a double-blind, randomized, placebo-controlled trial.	The plant extract was prepared as 1:1 in alcohol 45%.	In vivo	The extract produced a significantly better outcome on cognitive functions than placebo.	[40]
Lemon balm (*Mellia officinalis* L.)	Leaves	Rosmarinic acid (primary), flavonoids, phenolic acids	Iran	Alcoholic extract (60 drops/day); oral for 4 months.	The plant extract was prepared as 1:1 in 45% alcohol. The extract was standardized to contain at least 500 µg citral/mL.	In vivo	Cholinergic modulation (acetylcholinesterase inhibition).	[42]
		Rosmarinic acid	UK	Ethanolic extract (600 mg); oral, single dose, 7-day interval between each treatment condition.	Leaf extraction using a solvent and subsequent standardization.	In vivo	Nicotinic and muscarinic acetylcholine receptor binding and regulation.	[43]
*Ginger* (*Zingiber officinale*)	Rhizome	6-gingerol	China	6-gingerol (C17H26O4, purity ≥ 98%) (600, 1000, and 1600 mg of encapsulated dried leaf); directly to the culture medium; single administration.	Filtration, concentration, and solvent extraction (water or ethanol).	In vivo	6-gingerol may protect PC12 cells from Aβ1–42-induced apoptosis by lowering oxidative stress.	[44]
	Root	6-shogao	Japan	6-shogaol (10 mg/kg/day); oral.	Ginger rhizome extraction, followed by 6-shogaol isolation and purification.	In vivo	Reduction in neuroinflammation; suppression of the activation of microglia; decrease in proinflammatory cytokines; reduction in cognitive impairments.	[66]
Red ginger (*Z. officinale var. Rubra*) and white ginger (*Z. officinale var. Roscoe*)	Rhizome	Gingerol, shogaols, phenolic compounds	Nigeria	Aqueous extract (0–100 μL); directly added to rat brain tissue homogenates.	Before being ground, the edible portion was carefully cleaned with distilled water to get rid of any impurities, chopped into tiny bits, and allowed to air dry. The grinded samples were then soaked in water at 37 °C for approximately 24 h to create the rhizomes’ aqueous extract. The mixes were then filtered, and the filtrates were kept in the refrigerator for further examination.	In vitro	Inhibition of acetylcholinesterase (AChE).	[67]
Cinnamon bark (*Cinnamomum* spp.)	Bark	Phenylpropanoids	Korea	Methanolic extract (20; 50; 100 µM range); directly to the culture cells.	Bark is extracted using a solvent, and then phenylpropanoids are separated and fractionated.	In vitro	Cinnamon bark has the potential to be developed as an anti-Aβ treatment, which could in turn be beneficial for the prevention and treatment of AD.	[68]
Cinnamon (*Cinnamonum verum*)	Bark	Cinnamaldehyde, which is converted into cinnamic acid by oxidation	USA	Cinnamon extract and sodium benzoate (100 mg/kg body wt/d); oral, once daily/several months.	Metabolic conversion to sodium benzoate or direct administration of sodium benzoate.	In vivo	Enhancement of learning and memory.	[69]
Garlic (*Allium sativum* L.)	Bulbs	S-allylcysteine and allicin	Thailand	Ethanolic extract (125, 250, and 500 mg/kg); oral, once daily/several weeks.	The extract was prepared by soaking chopped garlic in 30% ethanol at a 1:3 ratio for approximately 15 months under light protection at room temperature.	In vivo	Cholinergic, glutamatergic, and GABAergic system modulation.	[70]
		S-allyl-cysteine	Korea	Ethanolic extract (5, 10, and 20 mg/kg of body); oral, once daily.	Long-term aging of garlic in an aqueous/ethanolic solution to preserve bioactive components.	In vivo	Antioxidant activities and neuroprotective effect against Aβ-induced cytotoxicity in neuron-like PC12 cells. Decrease in oxidative stress.	[71]
		Aged Garlic Extract and S-allyl-l-cysteine	USA	Aqueous aged garlic extract µM range, directly to the culture medium for 48 h (in vitro), oral feeding, 4 months (in vivo).	Aging of garlic in aqueous/ethanolic solution; isolation of S-allyl-l-cysteine.	In vivo/vitro	Extract treatment maintains the amounts of pre-synaptic proteins that determine pre-synaptic terminal structure and shields neuronal cells from oxidative insults caused by ROS.	[72]
Saffron (*Crocus sativus* L.)	Stigmas	Trans-crocetin	Italy	Purified trans-crocetin (5, 25, and 100 µM concentrations); directly to the culture medium; short-term incubation.	Extraction from saffron stigmas, followed by the hydrolysis of crocins and the purification of trans-crocetin.	In vitro/ex vivo	Increased breakdown of amyloid-β; Monocyte-mediated Aβ clearance activation.	[73]
	Stigmas	Crocins (trans-4-GG-crocin is the major crocetin-ester in the extract)	India	Hydroalcoholic extract—In vitro: 0.22–2.2 µg/mL extract (tightness and Aβ transport). In vivo: 50 mg/kg/day extract added to diet, oral administration, 1 month; Crocin alone tested at 10 mg/kg/day for 1 month.	Cold maceration of stigma in ethanol–water (1:1), repeated 3 times; extract characterized by HPLC/LC-MS.	In vitro In vivo	Increased production of tight-junction proteins and decreased permeability tighten the blood–brain barrier. The expression of the Aβ-degrading enzymes NEP and ABCA1 (ApoE clearance pathway) is increased. In 5XFAD brains, it decreases soluble and oligomeric Aβ.	[74]
	Dried stigma	Crocin, picrocrocin, and safranal.	Iran	Saffron capsules (30 mg/day); oral; once daily/12 months.	A total of 120 g of dried and milled *C. sativus* L. stigma was extracted with 1800 mL ethanol (80%) by percolation procedure in three steps, and then the ethanol extract was dried by evaporation at a temperature of 35–40 °C. Each capsule contained dried extract of saffron (15 mg), lactose (filler), magnesium stearate (lubricant), and sodium starch glycolate (disintegrant).	In vivo	One-year administration of saffron extract capsules was shown to be comparable with memantine in reducing cognitive decline in patients with moderate-to-severe AD.	[75]
Turmeric (*Curcuma longa*)	Rhizome	Curcumin	USA	Curcumic, a turmeric-derived compound; oral, 25 mg/kg and 50 mg/kg body weight; once daily/7 days.	Standard curcumin compound (purchased; not extracted in the study) 25 mg/kg and 50 mg/kg (in vivo). 100 µL of curcumin (0–5 µg/mL) compound (in vitro).	In vivo/vitro	Co-treatment with curcumin suppressed the increase in AChE activity and reduced AChE gene (mRNA) expression compared to Cd-only rats. Curcumin also directly inhibited rat brain AChE activity in vitro.	[76]
		Curcumin	Japan	Curcumin derivative known as FMeC1 (a curcumin analog with a substitution at the C-4 position on the molecule); dietary supplementation, continuous daily intake as part of diet/6 months.	Dietary inclusion of FMeC1 into mouse chow for chronic administration; curcumin and another analog (FMeC2) also tested as comparisons.	In vivo/vitro	More anti-amyloid pathology activity is demonstrated in vivo by the C-4 substituted derivative FMeC1 than by curcumin alone, indicating that structural alteration at C-4 improves therapeutic potential against Alzheimer-like amyloid pathology.	[45]
Ginkgo (*Ginkgo biloba*)	Leaves	flavonoid glycoside, terpenoids, organic acids	China	Standardized extract—EGb761 (50 mg/kg body weight); oral, once daily/6 months.	Standardized phytochemical extract that is sold commercially.	In vivo	In APP/PS1 mice, long-term EGb761 therapy enhances cognitive function and reduces amyloid pathology by boosting microglia surrounding the amyloid plaque and controlling inflammatory reactions.	[77]
		-	Germany	Standardized extract EGB 761; oral, once daily/23 days.	A total of 40 mg/tablet was prepared in drinking water (20 mg/mL).	In vivo/vitro	EGB 761 ameliorates the cognitive and memory impairment in a rat model of AD. The protective effects of HBO/EGB 761 are associated with reduced apoptosis and NF-κB pathway activation in hippocampus neurons.	[47]
			Germany	Dry extract (20 mg/kg^−1^/d^−1^); dietary supplementation; daily intake via diet/2–5 months.	Dry extract from Ginkgo (35–67:1) with an extraction solvent, acetone 60% (*w*/*w*), and adjusted to 22.0–27.0% ginkgo flavonoids, calculated as ginkgo flavone glycosides.	In vivo	Improved cognitive and memory function: Both EGB 761 and HBO significantly improved the performance of AD rats in the Morris water maze compared to model controls, with combined therapy demonstrating better results. Decreased apoptosis: Compared to the AD model control, TUNEL labeling revealed decreased hippocampus neuron apoptosis following EGB 761, HBO, and combination therapy.	[78]
Sweet wormwood (*Artemisia annua* L.)	Herbs	Artemisinin	China	Artemisinin (1 mg/kg; 5 mg/kg; oral, once daily (in vivo); SH-SY5Y cells: up to 200 μM artemisinin tested) directly to the culture medium (in vitro).	Standardized phytochemical extract that is sold commercially.	In vivo/vitro	ART significantly improved cognitive ability and improved neuronal functions by reducing oxidative stress and release of inflammatorily factors and apoptosis-related proteins and factors in 3xTg mice.	[79]
		Artemisinin B	China	Artemisinin B (98%) standardized (1, 2, 4 and 8 μM); oral, once daily.	Standardized phytochemical extract that is sold commercially.	In vitro/vivo	Artemisinin B improved spatial memory in dementia mice in the water maze and step-through tests, and altered the pathological features and the levels of inflammatory cytokines in the hippocampus and the cortex.	[80]
		Artemisinin	China	Dihydroartemisinin (DHA) (20 mg/kg/day); oral, once daily/3 months.	DHA was dissolved in 10% dimethyl sulfoxide (DMSO, Amresco, Solon, OH, USA) for oral treatment of mice.	In vivo	DHA alleviated memory deficits, decreased Aβ production and neuritic plaque formation, and ameliorated the autophagy flux in the brains of AD mice.	[81]

## Data Availability

The original contributions presented in the study are included in the article; further inquiries can be directed to the corresponding author.
